# Preparation of *β*-Myrcene-Chitosan Nanoparticles and Their Uptake and Toxicity in *Aedes aegypti* Larvae

**DOI:** 10.3390/insects15120998

**Published:** 2024-12-16

**Authors:** Lara T. M. Costa, Bruna L. Rocha, Cleidiane C. Teixeira, Hemilly C. Martins, Mauren Cristine A. Silveira, Benedito Albuquerque, Alex Sander R. Cangussu, Peng He, Raimundo Wagner S. Aguiar, Ana Maria S. Maia, Guy Smagghe, Eugênio E. Oliveira

**Affiliations:** 1 Departamento de Entomologia, Universidade Federal de Viçosa (UFV), Viçosa 36570-900, MG, Brazil; 2 Programa de Pós-Graduação em Biotecnologia, Universidade Federal do Tocantins (UFT), Gurupi 77402-970, TO, Brazilalexcangussu@uft.edu.br (A.S.R.C.);; 3 Departamento de Engenharia de Bioprocessos e Biotecnologia, Universidade Federal do Tocantins (UFT), Gurupi 77402-970, TO, Brazilanamaia@mail.uft.edu.br (A.M.S.M.); 4 Coordenação de Ciências Exatas e Biotecnológicas, Universidade Federal do Tocantins (UFT), P.O. Box 66, Gurupi 77402-970, TO, Brazil; 5 National Key Laboratory of Green Pesticide, Key Laboratory of Green Pesticide and Agricultural Bioengineering, Ministry of Education, Center for R&D of Fine Chemicals of Guizhou University, Guiyang 550025, China; phe1@gzu.edu.cn; 6 Institute of Entomology, Guizhou University, Guiyang 550025, China; 7 Department of Plants and Crops, Ghent University, 9000 Ghent, Belgium; 8 Department of Biology, Vrije Universiteit Brussel (VUB), 1050 Brussels, Belgium

**Keywords:** plant-based biorational pesticides, alternative mosquito control, histotoxicity

## Abstract

Mosquito control still relies heavily on synthetic molecules, which can lead to the selection of resistant populations and undesirable environmental problems. This study described the preparation of a nanoparticle of the plant-derived molecule, *β*-myrcene, with chitosan, and the assessment of its toxicity against larvae of the yellow fever mosquito, *Aedes aegypti*. By producing fluorescent chitosan nanoparticles, we were able to observe their distribution in the digestive tract of larvae of *Ae. aegypti*. Chitosan-based nanoparticles containing *β*-myrcene (238 mg/L) could kill 100% of the larvae tested, whereas the blank control (i.e., the nanoparticle without *β*-myrcene) showed no larvicidal activity. The chitosan nanoparticles with *β*-myrcene had a zeta potential of +15 mV and a hydrodynamic diameter ranging from 30 to 2800 nm. The blank control, without *β*-myrcene, had a zeta potential of +26 mV and a diameter of 30 to 830 nm. Fluorescence analysis showed that the nanoparticles were efficiently absorbed and distributed in the digestive tract organs of the *Ae. aegypti* larvae. In short, our results reinforce the benefits of using chitosan to carry molecules of plant-derived-molecules, such as *β*-myrcene, in mosquito control, suggesting a broad internal distribution that contributes to their toxicity.

## Simple Summary

The yellow fever mosquito, *Aedes aegypti*, is considered an important vector in the transmission of pathogens that cause major public health problems with diseases such as Zika, chikungunya, and dengue. The main current methods of controlling this vector rely on chemical products that favor the selection of resistant populations and can be harmful to human health and the environment (e.g., non-target organisms). It is, therefore, crucial to the development of novel approaches to control these pathogens vectors. Plant-based essential oil molecules, such as *β*-myrcene, have resurged as promising and more environmentally friendly alternatives for the better controlling of mosquitoes. However, due to the volatility of these compounds and their lipophilicity, their delivery and effectiveness can be compromised. Encapsulation in nanoparticles seems to be an effective solution for the stability and controlled release of these compounds. The nanoencapsulation of *β*-myrcene by chitosan helps to increase the effectiveness of this compound, maintaining a longer action and contributing to efficient distribution of *β*-myrcene in the target organism, favoring a more sustainable and effective pest control.

## 1. Introduction

The control of mosquitoes, including the yellow fever mosquito *Aedes aegypti*, is still massively dependent on the use of few synthetic molecules, and the misuses of such control tools has led to a selection of populations with multiple ways of resistance [1,2,3,4,5]. The emergence of insecticide resistance has been identified as one of the major factors, contributing to the persistence of epidemic diseases caused by mosquito-borne pathogens [3,6,7], which highlights the need to develop new interventions and approaches for controlling pathogen-transmitting mosquitoes [8,9,10,11]. Produced by plants, essential oils are volatile secondary metabolites (e.g., terpenes, terpenoids and molecules with aromatic groups) that have re-emerged as promising and environmentally safer approaches to be integrated in mosquito control [12,13,14].

The compound *β*-myrcene (7-methyl-3-methylene-1,6-octadiene) is an olefinic and acyclic monoterpene that occurs naturally in a large number of plant species, and its industrial uses comprise food additives, flavoring, cosmetics, soaps and detergents [15]. In addition, it has been identified as a potential alternative to be integrated in insect pest control programs. Previous studies evaluated *β*-myrcene as the main compound in the essential oil extracted from the leaves of neotropical plants such as *Siparuna guianensis*, which has been shown to be an alternative for controlling pathogens-vectoring mosquitoes and agricultural insect pests [16,17]. However, as demonstrated for several nonpolar and volatile molecules [13,18,19], it is necessary to use strategies to enhance the *β*-myrcene efficacy, which includes a proper carrier vehicle or nanoparticle.

The use of chitosan as a polymeric nanoparticle in the encapsulation of bioproducts has been presented as a promising carrier vehicle as it stabilizes, prevents degradation, and maintains the toxic effect of bioactive compounds for longer periods of time [20,21,22,23]. Chitosan is a linear-chain polysaccharide, composed of N-acetyl-D-glucosamine and D-glucosamine, and can be obtained from the deacetylation of chitin present in the exoskeleton of crustaceans, insects, and even in the cell walls of some fungi [24]. The high hydrophilicity of chitosan allows its use as a biomaterial in the form of a nanoparticle and as a drug delivery vehicle [25,26,27], and its uses in the encapsulation of essential oils has already been proved to maintain the insecticidal, antioxidant, antibacterial, and antifungal activities [20,21,28,29].

The solubility of novel plant-derived compounds often poses a challenge in animal experiments, as the agents used to dissolve these compounds frequently exhibit pharmacological properties that may influence the results [30,31]. Dimethyl sulfoxide (DMSO) is an amphipathic molecule with a highly polar domain and two apolar methyl groups, making it into one of the most common solvents used for the administration of water-insoluble substances [32]. Polysorbate 80 (Pol80) is a non-ionic surfactant widely used in the biopharmaceutical industry due to its high solubilization capacity and stability in water, as well as its biocompatibility with various products and low toxicity [33]. These characteristics help to form emulsions that prevent protein aggregation, improving the stability and efficacy of active compounds [34,35]. In this way, both Pol80 and DMSO can be an important factor in the formation of spherical nanoparticles, stabilizing on a smoother surface and facilitating the dispersion and efficacy of β-myrcene, as well as its stability in encapsulation, in addition to preventing the aggregation and self-association of bioactive compounds, resulting in a greater stability and efficiency in transport.

The gut or alimentary tract plays a crucial role in the biological functions in insects, encompassing the initial breakdown of food, nutrient absorption, and waste excretion [35,36,37]. Despite the fact that the alimentary tract of insects has served as the major target for bacterial entomopathogens and their toxin-based products [38,39,40], the damage caused in the alimentary canal organ and digestive system has been shown to potentiate the actions of synthetic insecticides [41,42]. These insect physiological organs and processes have been targeted by plant-based molecules alone or encapsulated in nano- and micro-structures [22,43,44,45,46,47].

Here, we report on the preparation of chitosan nanoparticles with *β*-myrcene and their uptake and toxicity in *Aedes aegypti.* Specifically, using the nanoencapsulation of *β*-myrcene with fluorescent chitosan molecules, we assessed whether these approaches would reduce toxicity losses related to the volatility and lipophilicity of *β*-myrcene, but also to evaluate whether chitosan nanoparticles containing *β*-myrcene were absorbed and distributed along internal tissues of the mosquito larvae alimentary tract.

## 2. Materials and Methods

### 2.1. Aedes aegypti Larvae and Reagents

Third instar larvae of *Ae. aegypti* were obtained from a mixed colony of individuals originally collected in the county of Palmeirópolis (latitude: 13°2′36″ South, longitude: 48°24′11″ West) Tocantins State, Brazil, that had been reared under controlled conditions (SISGEN registration number A6C82F8) at the Laboratory of Molecular Biology of the Federal University of Tocantins (UFT, Campus of Gurupi, Gurupi, Tocantins State, Brazil). Rearing was conducted according to already published methodology [11]. The adult mosquitoes were kept in acrylic cages with water jars containing paper for collecting eggs, and vials containing a 10% aqueous sucrose solution and blood. The larvae were raised in plastic containers (35 cm × 5 cm) and fed with turtle food (Reptolife, Alcon Pet, Camburiú, SC, Brazil). Rearing and bioassays were conducted at 26 ± 1 °C, 60 ± 5% RH, with a 12 h light–dark photoperiod. The *β*-myrcene (technical grade), chitosan $\bar{Mv}$ 4.9 × 10^4^ g/mol and deacetylation degree of 76.5%, sodium tripolyphosphate (TPP) (KF = 0.2%), and fluorescein isothiocyanate (FITC) (99%) were obtained from Sigma-Aldrich Brasil, Cotia, SP, Brazil. All chemicals were of analytical grade and used without further purification. All analyses were performed with distilled water.

### 2.2. Assessment of Acute Toxicity of β-Myrcene Against Aedes aegypti Larvae

Firstly, we conducted a preliminary test in order to determine the minimum concentration of Pol80, as a solubilizing agent, capable of homogenizing *β*-myrcene in an aqueous medium. The Pol80 solutions (10–100 µM) were prepared by dissolving Pol80 in 1 L volumetric flasks. The samples were gently shaken until the complete dissolution of Pol80. The 20 µM solution was considered adequate for further testing. Acute toxicity bioassays were performed as previously described elsewhere [44,45]. We compared the larval mortality results obtained using Pol80 as a solubilizing solvent with the results obtained using DMSO at a concentration of 211 µM. Groups of 25 larvae were placed in glass beakers with 30 mL of the Pol80- or DMSO-containing solution. An aqueous solution of the solubilizing agent and distilled water were used as controls. The number of dead larvae was quantified every 15 min in the first two hours of exposure, and they were considered dead when unable to move or to respond to touch stimuli with a Pasteur pipette. For each concentration, four repetitions were made. The percentage of larval mortality was corrected for natural mortality observed in the control treatment using Abbott’s formula before being analyzed.

### 2.3. Preparation of Nanoparticles

The nanoparticles were prepared using a modified ionic gelation technique as previously described elsewhere [46,47,48]. Briefly, we dissolved 0.5 g of chitosan (CHIT) in 200 mL of acetic acid 2% (*v*/*v*), and the solution was vacuum filtered with the aid of an LT65 vacuum pump (Lima Tec Equipment, Cariacica, ES, Brazil) in a P1 filter. We added 2 g of Pol80 to the CHIT solution and the mixture was kept under magnetic stirring for 2 h, at 45 °C, in order to obtain a 1% (*m/v*) surfactant concentration. The pH of the solution was corrected to 4.5 with 0.1 mol/L NaOH, which was slowly dropped into the stirred solution. The *β*-myrcene was added with stirring for 10 min, in order to obtain a *β*-myrcene concentration equal to 300 mL/L (i.e., 0.03% (*v*/*v*). To carry out the crosslinking of CHIT to obtain CHIT nanoparticles with *β*-myrcene (CNPM), a solution of TPP in the ratio of 3:0.8 (CHIT: TPP) was prepared. The TPP solution slowly dripped at a height of 8 cm into the CHIT solution. The pH was corrected to 7.0 with NaOH 0.1 mol/L under gentle stirring. After the nanoparticles were centrifuged at a 1811 relative centrifugal force (RCF) for 15 min, simultaneous washings with distilled water to remove free TPP [49] was performed. For the preparation of CHIT nanoparticles without *β*-myrcene (CNPP), the same protocol was used, with no addition of *β*-myrcene. Samples were stored at 4 °C until the FITC binding step.

Chitosan nanoparticles containing *β*-myrcene (CNPM) were redispersed in 50 mL of distilled water with magnetic stirring for 2 h, and 50 mL of methanol was slowly added to that suspension. Then 50 mL of a 50% FITC solution in methanol was slowly added into the CNPM suspension under intense stirring. The system remained under stirring for 2 h, in the absence of light and at room temperature. After this period, it remained at rest for 24 h. Afterward, the nanoparticles underwent centrifugation and were washed with methanol to eliminate free FITC. This process ran until FITC absence was confirmed in the supernatant via UV-Vis spectrophotometry. Measurements were conducted using a UV-VIS spectrophotometer model T 70 from PG INSTRUMENTS, with a scanning range of 400–800 nm and utilizing 1 cm quartz cuvettes [50]. The samples were redispersed in distilled water, remaining under stirring for 12 h to remove methanol (by evaporation), with precautions taken to prevent light exposure. The same procedure was performed with the CNPP.

### 2.4. Nanoparticle Characterization

We transferred 10 mL of the colloidal suspension to an electrophoretic cell and recorded the zeta potential values using the Stabino II software (Colloid Metrix, Meerbusch, Germany). Data were collected at room temperature and treated with the Stabino Control software (v. 2.00.27.02). To obtain the morphology and size of the particles, the sample was redispersed in water with ultrasound, placed on the stub and dried in an oven for 4 h at 37 °C. The particles were covered with gold for 180 s using an Emitech instrument (model K550) and observed in a Zeiss scanning electron microscope (Model DSM 962) at 15 kV. Infrared spectra (IR) were performed in a Perkin Elmer spectrophotometer (model Spectrum Two) using KBr discs. Samples were analyzed between 4000–400 per cm.

### 2.5. Fluorescence Microscopy Analysis of Mosquito Larvae Exposed to Nanoparticles

We adjusted the suspension volumes to 200 mL with distilled water. Then, 30 mL of each of the resulting suspensions were placed in contact with groups of 25 *Ae. aegypti* larvae. After 24 h of exposure, 5 larvae were randomly collected from each treatment to prepare slides for fluorescence microscopy. The slides were prepared as described elsewhere [51,52]. The selected larvae were subjected to the following procedures: fixation, dehydration, diaphanization, inclusion in paraffin, molding of the blocks, and microtomy. The paraffin blocks were cut in a manual rotating microtome, adjusted to provide 3 µm thick cuts. The slides were observed under a Zeiss inverted fluorescence microscope, Axiovert 100, with a 580 nm filter.

### 2.6. Toxicity of β-Myrcene-Containing Nanoparticles Against Aedes aegypti Larvae

The bioassays conducted to assess the toxicity of chitosan nanoparticles containing β-myrcene followed the same procedures described above for acute toxicity of β-myrcene. We subjected groups of 25 larvae to 30 mL of a solution that had chitosan nanoparticles containing β-myrcene at a concentration of 238 mg/L. The blank control consisted of a solution containing nanoparticles without β-myrcene. The exposure period was 2 h, when we recorded the number of dead larvae. For each treatment, we used four repetitions. The percentage of larval mortality was corrected for natural mortality observed in the control treatment using Abbott’s formula before being analyzed.

### 2.7. Statistical Analysis

Comparisons of the results for the mortality of *Ae. aegypti* larvae under exposure of formulations with TPP or DMSO/were conducted using Tukey’s HSD test, considering significant differences for *p* < 0.05, available in SigmaPlot 12.0 (Systat Software, San Jose, CA, USA).

## 3. Results

### 3.1. Nanoparticles Containing β-Myrcene Kill Mosquito Larvae

**Figure 1:**
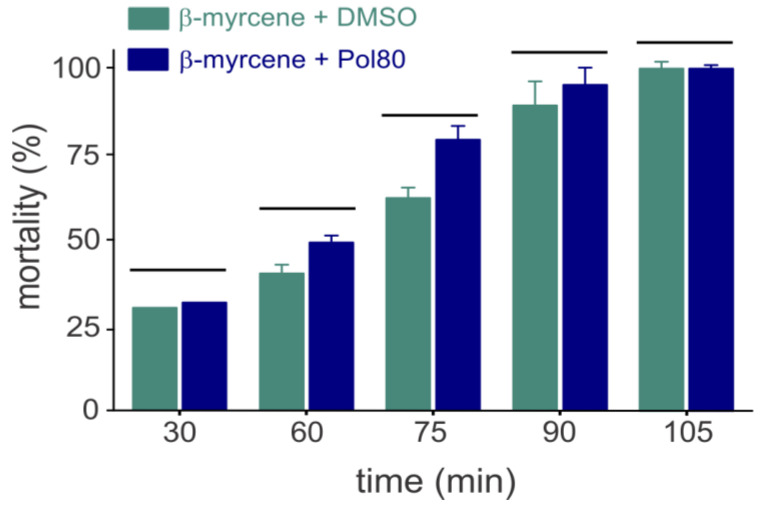
Toxicity of *β*-myrcene against *Aedes aegypti* larvae using dimethyl sulfoxide (DMSO) or polysorbate 80 (Pol80) as solubilizing agents. The *β*-myrcene concentration was of 238 mg/L. Each bar represents the mean (±standard error) of four replicates. Bars grouped by horizontal lines indicate the absence of significant statistical differences (Tukey’s Honest Significant Difference (HSD) test, *p* > 0.05).

The toxicity of *β*-myrcene (238 mg/L) against the mosquito larvae was not affected (*p* > 0.05) by the solubilizing agent types, i.e., of Pol80 or DMSO (Figure 1). Nanoparticles containing β-myrcene (238 mg/L) did kill 100% of the larvae, while the blank control (i.e., the nanoparticle without β-myrcene) caused no mortality.

### 3.2. Characterization of the Nanoparticles

**Figure 2:**
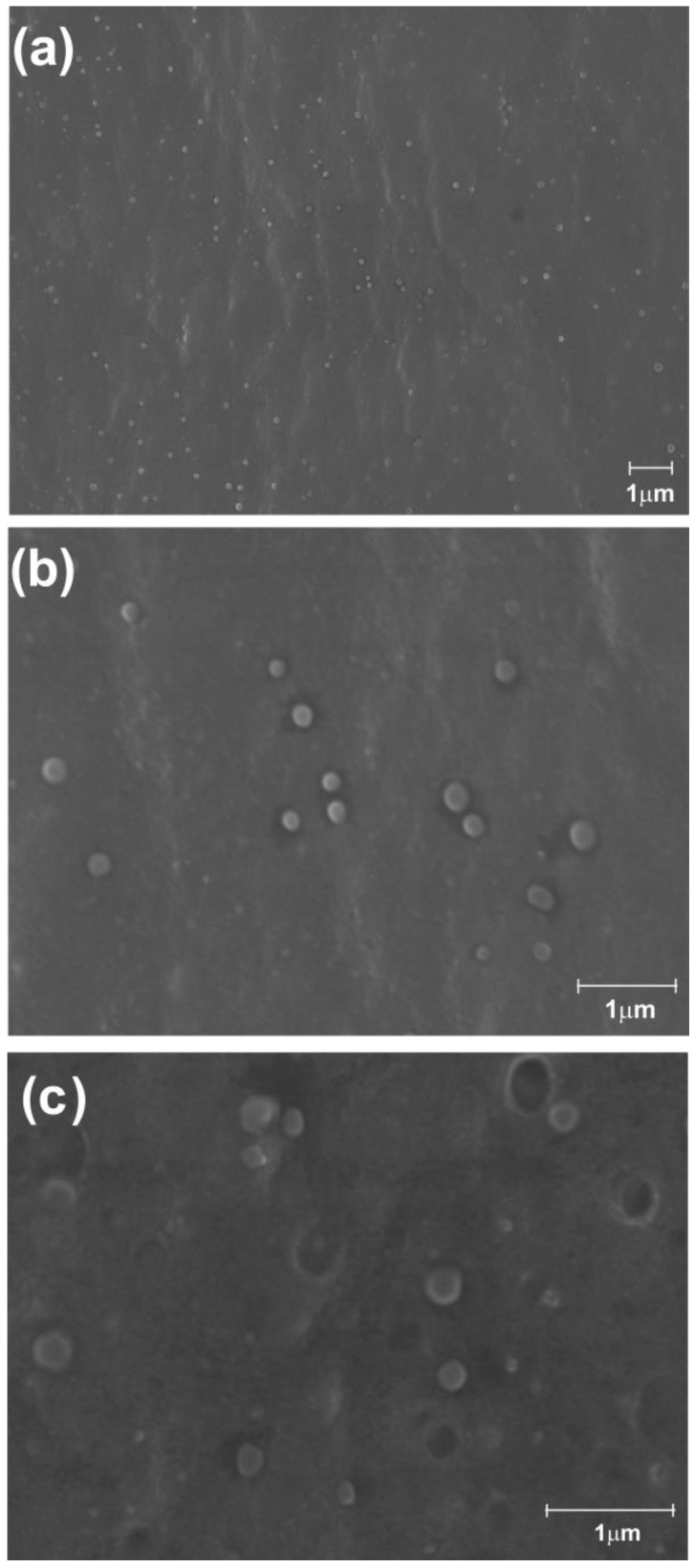
Scanning electron microscopy (SEM) of chitosan nanoparticle suspensions, containing β-myrcene, at a magnification of 4500× (**a**), 18,000× (**b**), and 22,000× (**c**). The nanocomposites exhibited a spherical structure with a smooth surface and showed a size variation ranging from 30–280 nm.

The zeta potential on the surface of the nanoparticles was determined to be +14.8 mV, from β-myrcene-containing nanoparticles and +26.5 mV from nanoparticles without β-myrcene, and the myrcene-containing nanoparticles were analyzed by scanning electron microscopy (SEM) (Figure 2).

**Figure 3:**
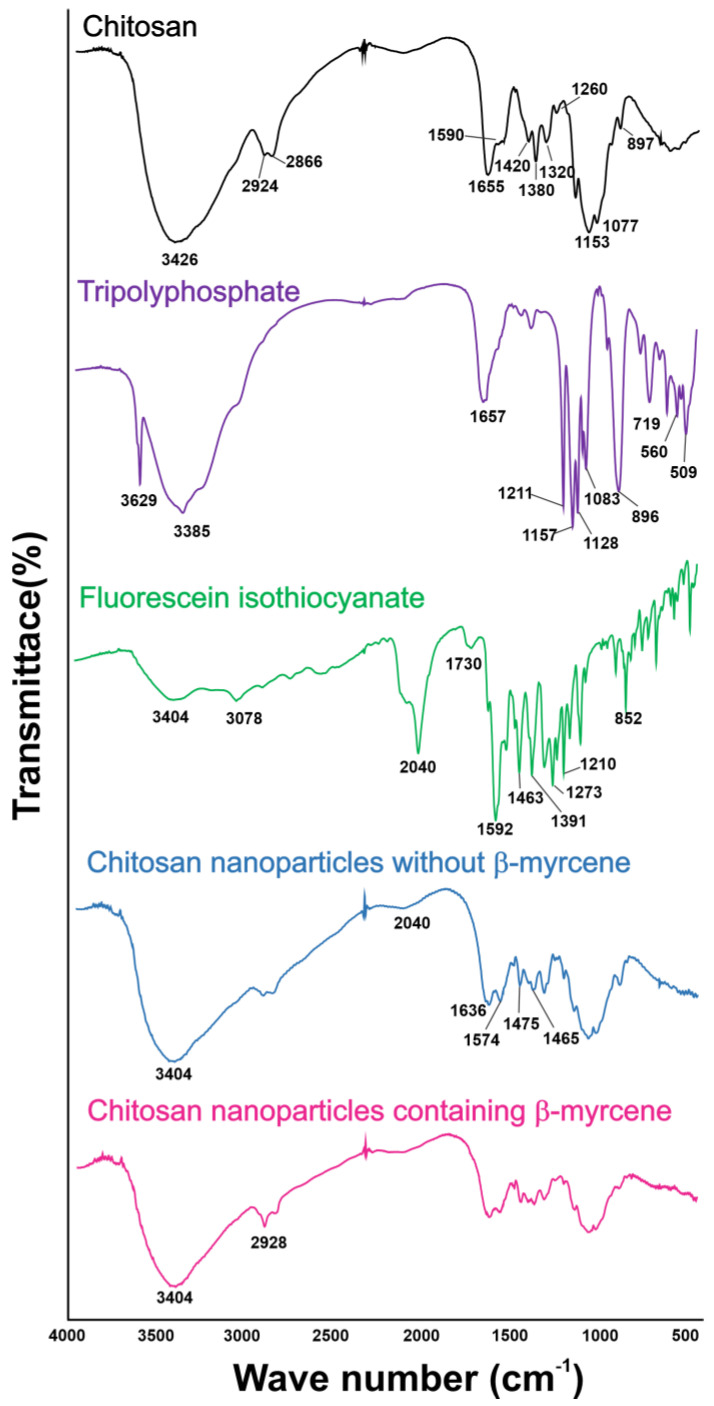
Fourier transform infrared (FTIR) spectra of chitosan nanoparticle (black), tripolyphosphate (TPP) (purple), fluorescein isothiocyanate (FITC) (green), chitosan nanoparticle without β-myrcene (blue), and chitosan nanoparticle containing b-myrcene (magenta). For an interpretation of the references to color in this figure legend, the reader is referred to the electronic version of this article.

As described in Figure 3, the Fourier transform infrared (FTIR) analysis of chitosan revealed the band corresponding to ν_C=O_ of amide (amide I) and δ_N–H_ of amide (amide II) at 1655 per cm and 1590 per cm (Figure 3, black line). The spectrum for TPP is shown in Figure 3 (purple line). Bands at 3629 per cm and 3385 per cm are related to νO–H. At 1657 per cm is the band related to δ_O–H_. The peaks at 1211, 1157, and 1128 per cm are attributed to the asymmetric νPO_3_^2−^ of the terminal groups. The peak at 1083 per cm is due to the symmetrical deformation of the terminal PO_3_^2−^ groups. The peaks at 896 and 719 per cm are characteristic of the asymmetrical and symmetrical axial deformation, respectively, of the main-chain P-O-P bond. The peaks at 560 and 509 per cm may be attributed to the δ PO_3_^2−^ of the terminal groups [53]. The FTIR analysis for FITC revealed low-intensity bands at 3404 per cm, referring to the ν_O–H_ bond and at 3078 per cm, referring to the ν_C–H_ bond of the aromatic ring (Figure 3, green line). In 2040 per cm, there is an intense band referring to the N=C=S group [54]. Chitosan nanoparticles without β-myrcene (Figure 3, blue line) and with β-myrcene (Figure 3, magenta line) showed the following bands: 1633, 1574, 1463, and 1475 per cm, which correspond to ν_C–H_ of amide (amide I), δ_N–H_ of amide (amide II), C=C from aromatic ring, and N-H from amine, respectively.

### 3.3. Fluorescence Microscopy

**Figure 4:**
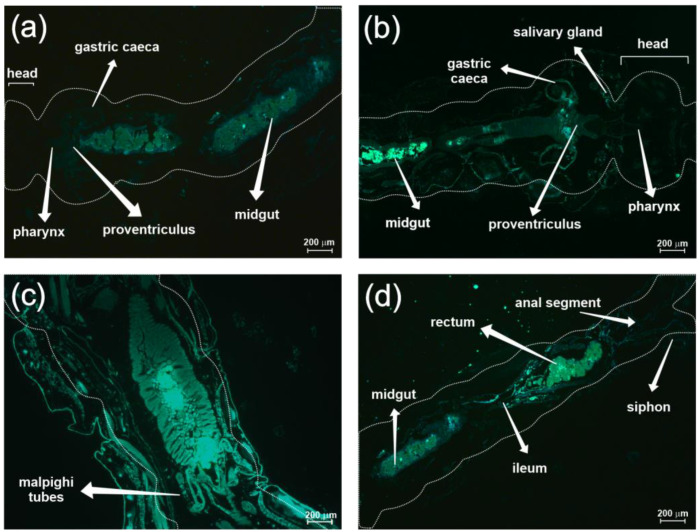
Fluorescence tracing of chitosan nanoparticles in the digestive system of mosquito larvae. (**a**) Head, gastric caecum, midgut, proventriculus, and pharynx of larva treated with chitosan nanoparticle without β-myrcene (CNPP), at 5× magnification. (**b**) Head, salivary gland, caecum, midgut, proventriculus, and pharynx of larva treated with chitosan nanoparticle with β-myrcene (CNPM), at 5× magnification. (**c**) Midgut, rectum, midgut, ileum, anal segment, and siphon, of larva treated with CNPP, at 20× magnification. (**d**) Midgut of larva treated with CNPM, at 5× magnification. The dashed lines represent the exoskeleton of the larvae.

The images obtained with the fluorescence microscope revealed that CNPM and CNPP nanoparticles (i.e., containing and not containing β-myrcene) could be widely distributed in the alimentary tracts of the larvae (Figure 4). However, intense fluorescence was detected for the foregut, midgut, and hindgut sections.

## 4. Discussion

Here, we demonstrated the preparation of β-myrcene-chitosan nanoparticles with *β*-myrcene and the efficacy of these nanoparticles in targeting mosquito larvae, particularly *Ae. aegypti*. We further investigated the influence of solubilizing agents, namely DMSO or Pol80, on the efficacy of *β*-myrcene and found that its actions were not contingent upon the solubilizing agent used. Furthermore, engineered fluorescent nanoparticles encapsulating the *β*-myrcene within chitosan matrices demonstrated that these nanoparticles efficiently disperse throughout the alimentary tract of *Ae. aegypti* larvae, which may have contributed to the larvicidal activities of *β*-myrcene. Mosquito larvae, known to be filter feeders that extract particulate matter from the water to feed, are potential targets for nanoparticle-based interventions [55].

The use of natural products in pest control has become a significant alternative for replacing or being integrated in the biorational management of pest organisms [56,57,58,59]. However, recent investigations have shown that these biorational molecules, derived from plants, pose some challenges due to their rapid degradability and poor water solubility for larvicidal use [9,12,60]. Thus, the encapsulation of *β*-myrcene in colloidal systems emerges as a promising strategy for potential applications in vector control programs, both to stabilize the compound’s properties and to facilitate its administration in target environments (i.e., aquatic ecosystems that facilitate the development of mosquito larvae).

Our results with the Pol80 as a solubilizing agent of β-myrcene revealed this compound as a promising replacement to DMSO in the development of products aiming to control mosquito larvae. Pol80 exhibits a high solubilizing capacity and stability in water and showed neutrality in the toxicity of β-myrcene to *Ae. aegypti* larvae, a crucial consideration for larvicidal formulations [20]. Furthermore, by loading the β-myrcene-containing nanoparticle with chitosan, we obtained spherical nanoparticles with a smooth surface and approximate size of 250 nm, as already observed for other chitosan nanoparticles cross-linked with TPP and containing essential oils [61]. The formation of such nanoparticles was mediated by the electrostatic interaction between protonated amino groups (NH^3+^) of positively charged chitosan and phosphate ions (P_3_O_10_^5−^) from the TPP with negative charge [50].

Although *β*-myrcene has been described as a predominant component in several essential oils, the insecticidal efficacy of *β*-myrcene in its pure form has not been investigated in detail. These oils have demonstrated effectiveness against mosquitoes and other pathogens vectors [38,62,63,64,65]. For instance, previous investigations have shown that an essential oil consisting of 79.7% of *β*-myrcene exhibited notable toxicity against larvae of *Ae. aegypti* with an LC_50_ value of 0.98 µg/mL [45]. It is worth noting that although the essential oil is primarily β-myrcene, its toxicity is approximately 240-fold greater than the results described in our manuscript, which suggests that the vehicle or other essential oil components, even at a lower concentration, is contributing to the essential oil toxicity. Other investigations with essential oils consisting of lower quantities (varying from 7.8% up to 10.5%) of *β*-myrcene were also able to show larvicidal activities against *Ae. Aegypti*; however, they performed with less potency (LC_50_ varying from 47.2 μg/mL up to 163.8 μg/mL [64,65,66]. These findings highlight *β*-myrcene as a potential insecticidal tool, particularly for the control of mosquito vectors.

Our analysis for the nanoparticle’s properties revealed discrepancies in the values of zeta potential and hydrodynamic diameters between the nanoparticles containing *β*-myrcene and those lacking *β*-myrcene in their constitution. Such differences in these values may have resulted out of a *β*-myrcene presence on the nanoparticle surface, leading to the formation of larger aggregates, which potentially altered its structure and interactions with the polymeric matrix, indicating a greater instability [67]. The presence of a positive zeta potentially facilitates its interaction with negatively charged cellular surfaces, leading to an increased cellular uptake. Thus, positively charged nanoparticles, such as those containing chitosan, may adhere more easily to the insect mucosa and transiently disrupt tight junctions between epithelial cells, favoring their absorption [68]. By increasing the cellular uptake, chitosan nanoparticles can favor the uptake and effectiveness of several therapeutic and insecticidal molecules [20,69,70].

Fluorescence microscopy images provided insights into the distribution and localization of chitosan nanoparticles throughout the alimentary tract of the mosquito larvae. Here, we further demonstrated that the formation of fluorescent chitosan nanoparticles with and without *β*-myrcene caused a shift of the chitosan-related amide I (from 1655 per cm to 1633 per cm) and II (from 1694 per cm to 1574 per cm) bands, indicating interactions between them. Further support to this is provided by the appearance of a band at 1463 per cm, associated with the deformation of the C=C bond of the aromatic ring of fluorescein, confirming the presence of *β*-myrcene within the nanoparticle. Similarly, the increase in intensity of the band at 2928 per cm, with the incorporation of β-myrcene, suggested alterations in molecular vibrations, possibly explained by interactions between the *β*-myrcene and chitosan molecules.

Confirmation of covalent binding with fluorescein is given by the absence of the band at 2040 per cm, characteristic of the isothiocyanate group, and the appearance of a band at 1475 cm, which can be attributed to the secondary amine N-H bond [71]. As stated earlier, the significant distribution of ingested nanoparticles suggests an efficient absorption and transport along the larvae alimentary canal, attributed to various factors, including the positive charge of the nanoparticle, as indicated by their positive zeta potential. Indeed, the uptake in mosquitoes, in their cells and tissues, is important to explain the activity of biological compounds [72,73]. Positively charged nanoparticles have a higher affinity for mucosal surfaces and can transiently disrupt tight junctions between epithelial cells, facilitating their absorption [64]. Furthermore, previous investigations [74] have shown that electrostatic interactions between positively charged nanoparticles and negatively charged plasma membranes can lead to tissue barrier breakdown and promote accumulation in various organs

## 5. Conclusions

Here, we have demonstrated the preparation of chitosan nanoparticles with β-myrcene and the efficacy of these to kill *Ae. aegypti* larvae. This confirms the contribution to the larvicidal activity demonstrated by essential oils such as *S. guianensis*, but we also showed that β-myrcene-mediated toxicity is not dependent on the solubilizing agent used. Furthermore, our findings reinforce the idea that the development of nanoparticle-based products can efficiently work in the management of mosquito larvae, especially due to the fact that these individuals are filter feeders that extract particulate matter from the water to feed. The β-myrcene-containing chitosan-loaded nanoparticles were absorbed and distributed in several alimentary tract organs in the Ae. aegypti larvae, as observed by fluorescence microscopy, suggesting that the nanoparticle increases the uptake of β-myrcene by facilitating its ingestion and absorption by the larvae and so in turn increases its larvicidal activity. These β-myrcene-containing chitosan-loaded nanoparticles strategies offer a promising avenue for effective mosquito control.

## Data Availability

All the data is contained in the article and can be made available in the event of requests.
